# Buffalo Medical College

**Published:** 1850-03

**Authors:** 


*Buffalo Medical College. Commencement.*—We copy the following notice of the annual commencement in this Institution, on the 27th ult. from the Daily Commercial Advertiser of this city:

"The Annual Commencement of the Medical Department of the University of Buffalo, took place yesterday afternoon at Clinton Hall, in the presence of a very large audience of ladies and gentlemen.

"The exercises were opened with music by the band, which was in attendance, followed with prayer by Dr. Thompson, of the First Presbyterian Church.

"The Degree of M.D. was then conferred upon the graduating Class—twenty-seven in number—by Hon. G. W: Clinton, in the absence of the Chancellor, Hon. M. Fillmore. After conferring the Degrees, Mr. Clinton addressed the graduates briefly, but eloquently, in behalf of the Council.

"The following are the names of the graduating Class:

Emiel J. Dahm, Rochester.

William Gould, Pekin.

James S. Hawley, Camillus.

Hugh B. Van Deventer, Buffalo.

John A. Morse, Constantine, Mich.

Alfred M. Leonard, Lockport.

Orrin A. Lovejoy, Newark.

Leonard G. Hall, Penfield, Va.

William Thorne, Sinclearville.

Edwin G. Bly, Buffalo.

Lovinus F. Hillman, Parma.

Peter B. Brown, Somerset.

Thomas Burns, Illinois.

Byron De Witt, Auburn.

Hugh McKennan, Middleport.

Charles Jewett, Moravia.

John Root, Sweden, N. Y.

John E. Ware, C. W.

Clinton Colegrove, Sardinia.

George Johnson, Black Rock.

Matthew F. Haney, St. Johns, C. W.

George A. Hewson, Penn Yan.

Alfred H. Robbins, Logansport, Ia.

Warham B. Williams, Hallsburg, Pa.

Samuel B. Brinkerhoff, Auburn.

Charles E. Van Anden, Auburn.

William Hyser, Buffalo.

"The Honorary Degree of M.D. was conferred upon Samuel Carey, of this city.

"Next followed the address to the graduates, by Prof. J. P. White, which was a very appropriate and well written production—full of good advice to the young gentlemen who are just entering upon a career of active duties, and of valuable suggestions for their guidance in their future course.

"The exercises were concluded with the benediction by the Rev. Dr. Shelton.

"The College is in a very flourishing and satisfactory condition. About

120 students were in attendance during the past term. The course pursued by the Faculty, on account of the thoroughness and completeness of the instruction given, and the attainments required for graduation, is calculated to give the institution a permanent reputation, which must continue to increase with each annual session. Its friends have every reason to be satisfied and gratified with the results thus far, and with the promises of the future."

